# The vaginal microbiota in the course of bacterial vaginosis treatment

**DOI:** 10.1007/s10096-020-04049-6

**Published:** 2020-10-07

**Authors:** Romy D. Zwittink, Ellen H. A. van den Munckhof, Maurine A. Leverstein-van Hall, Kim Boers, Anco Molijn, Cornelis W. Knetsch, Ed J. Kuijper

**Affiliations:** 1 Center for Microbiome Analyses and Therapeutics, Leiden University Medical Center, Leiden, The Netherlands; 2 Department of Medical Microbiology, Leiden University Medical Center, Albinusdreef 2, 2333 ZA Leiden, The Netherlands; 3 DDL Diagnostic Laboratory, Rijswijk, The Netherlands; 4 Department of Medical Microbiology, Alrijne Hospital, Leiden, The Netherlands; 5 Department of Gynaecology, Haaglanden Medical Centre, The Hague, The Netherlands

**Keywords:** Antibiotics, Bacterial vaginosis, Clindamycin, Metronidazole, Microbiota, 16S rRNA gene amplicon sequencing

## Abstract

Bacterial vaginosis (BV) is perceived as a condition of disrupted vaginal microbiota, but remains of unknown aetiology. In this study, vaginal microbiota composition was determined in twenty-one women with BV, before and after treatment with metronidazole or clindamycin. Microbiota composition varied greatly between women and defining a (un)healthy vaginal microbiota state remains elusive, challenging BV diagnosis and treatment. While relative abundance of *Lactobacillus* increased after antibiotic treatment in two-third of women, its abundance was not associated with treatment outcome. Instead, remaining complaints of abnormal vaginal discharge were more common after metronidazole treatment and associated with increased relative abundance of *Ureaplasma*.

## Introduction

The vaginal microbiota plays a crucial role in maintaining a healthy vaginal environment, and perturbation of this system has been implicated in disturbed vaginal health and other negative outcomes [1, 2]. The vaginal microbiota is dynamic and influenced by hormonal changes, sexual activity, and hygiene [3]. Various vaginal bacterial communities exist in healthy women, mostly dominated by *Lactobacillus* species, while some are being composed of anaerobes like *Atopobium* and *Prevotella* species [4]. Nevertheless, the common perception of a healthy vaginal microbiota is one dominated by one or more *Lactobacillus* species. As such, the switch from a *Lactobacillus*-dominated microbiota to a more diverse microbiota, in combination with clinical symptoms, is considered bacterial vaginosis (BV) or aerobic vaginitis, depending on colonisation by anaerobic or aerobic bacteria, respectively. Bacterial genera that are specifically associated with bacterial vaginosis are, amongst others, *Gardnerella*, *Atopobium*, *Prevotella*, *Fusobacterium*, and *Dialister* species [5]. Despite these associations, the aetiology of BV is unknown, and diagnosis and treatment remain elusive. While a Gram-stain evaluation according to the Nugent criteria is considered the golden standard for BV diagnosis, it is not routinely applied in a clinical setting [6]. Instead, BV diagnosis is commonly based on clinical signs and symptoms or Amsel criteria [7]. Symptoms of BV can be resolved without intervention, but metronidazole or clindamycin can be prescribed in case of persistence, even though recurrence is common [8, 9]. In our study, vaginal microbiota composition of women with clinically diagnosed BV was determined before and after antibiotic treatment and related to clinical characteristics.

## Materials and methods

Prospectively, vaginal secretions and clinical data were collected from 60 premenopausal women visiting the gynaecology outpatient clinic of the Haaglanden Medical Centre (The Hague, The Netherlands) with complaints of abnormal vaginal discharge. Vaginal secretion was collected using the ESwab (Copan Diagnostics Inc., USA). BV diagnosis was based on clinical symptoms and signs, with malodorous discharge, as major criteria for diagnosis of bacterial vaginosis, followed by culturing when clinical diagnosis based on symptoms alone was uncertain. Therapy was initiated according to routine hospital practice following the European guideline and consisted of 500 mg metronidazole taken orally twice a day for 7 days, or, in case of pregnancy or lactating, 300 mg clindamycin taken orally twice a day for 7 days [10]. A follow-up visit was scheduled approximately 4 weeks after inclusion, during which vaginal swab and clinical data collection were repeated. Women who were clinically diagnosed with BV and attended the follow-up visit were selected for microbiota profiling (*n* = 21). Clinical data collection, Amsel criteria (vaginal pH, amine odour, wet-mount microscopy), Nugent score, and *Gardnerella vaginalis* culturing were performed for research purposes as previously described [11]. Detailed subject characteristics are outlined in Table 1. The Declaration of Helsinki was the guiding principle for trial execution, and the study was approved by the local ethics board (METC Zuidwest Holland, The Hague, The Netherlands). All patients provided written informed consent before participation.

**Table 1:** Subject characteristics

	Before treatment	After treatment
Demographics		
*n*	21	21
Age (years)	32.5 ± 7.6	32.5 ± 7.6
European	15	15
Antimicrobials		
Clindamycin	-	11
Metronidazole	-	10
Clotrimazole	-	4
Azithromycin	-	1
Symptomology		
Abnormal discharge	21	9
Malodorous discharge	20	4
Increased discharge	13	5
Yellow/green discharge	7	2
Curdy discharge	2	2
Thin white discharge	8	5
Purulent discharge	1	0
Vulvar erythema oedema	4	2
Vulvar itching	9	3
Vulvar irritation	6	3
Cervical erythema	3	2
Cervical bleeding	1	0
Low abdominal pain	10	3
Diagnosis		
Bacterial vaginosis	21	2
Nugent score positive	12	5
Amsel criteria positive	13	4
Vaginal pH > 4.5	18	12
Amine odour	16	8
Clue cells	14	4
Other		
Anticonception	6	6
Vaginal shower gel	0	1
Sexually active	20	20
Pregnant	8	8
Lactating	3	3
PROM	0	0

Vaginal bacterial microbiota was determined by 16S rRNA gene amplicon sequencing of the V3-V4 region using the Nextera XT, MiSeq Reagent Kits v2 500 cycles, and a MiSeq desktop sequencer (Illumina, USA). Raw sequencing data are available in the NCBI Sequence Read Archive (https://www.ncbi.nlm.nih.gov/sra) under study accession PRJNA524112. Read filtering, operational taxonomic unit (OTU)-picking, and taxonomic assignment were performed using the NG-Tax 0.4 pipeline and the Silva_132_SSU Ref database [12]. Statistical analysis and data visualisation were performed in R (v3.5.1) using the packages phyloseq (v1.26.1), vegan (v2.5-4), ggplot2 (v3.1.0), DESeq2 (v1.22.2) microbiome (v1.4.2), and DirichletMultinomial (v1.24.1). For differential abundance testing by DESeq2, the OTU-table was filtered for OTUs present in less than 25% of the samples to minimise zero-variance errors and spurious significance. Permutational multivariate analysis of variance was performed using the adonis function with 999 permutations and Bray-Curtis distances to determine associations between microbiota composition and clinical variables. The Dirichlet Multinomial Mixtures method, using the Laplace equation, was applied for community typing. In this approach, samples are clustered based on microbiota profile similarity [13]. Kruskal-Wallis followed by post hoc Dunn’s testing was performed to compare Shannon diversity indices between groups.

## Results and discussion

**Fig. 1:**
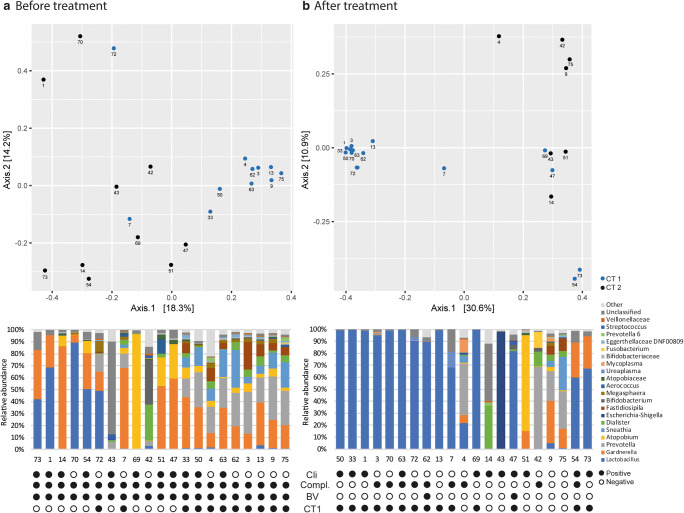
Principal coordinate analysis and taxonomic profiles of the vaginal microbiota before (**a**) and after (**b**) antibiotic treatment. Numbers indicate individual patients. Twenty taxa with highest average relative abundance are shown; abundances of all other taxa are summed and categorised as ‘other’. For bar graphs, the subject order is matched to the subject order in the PCoA plots. Cli, clindamycin; Compl., complaints of abnormal vaginal discharge; CT1, community type one; CT2, community type 2

**Fig. 2:**
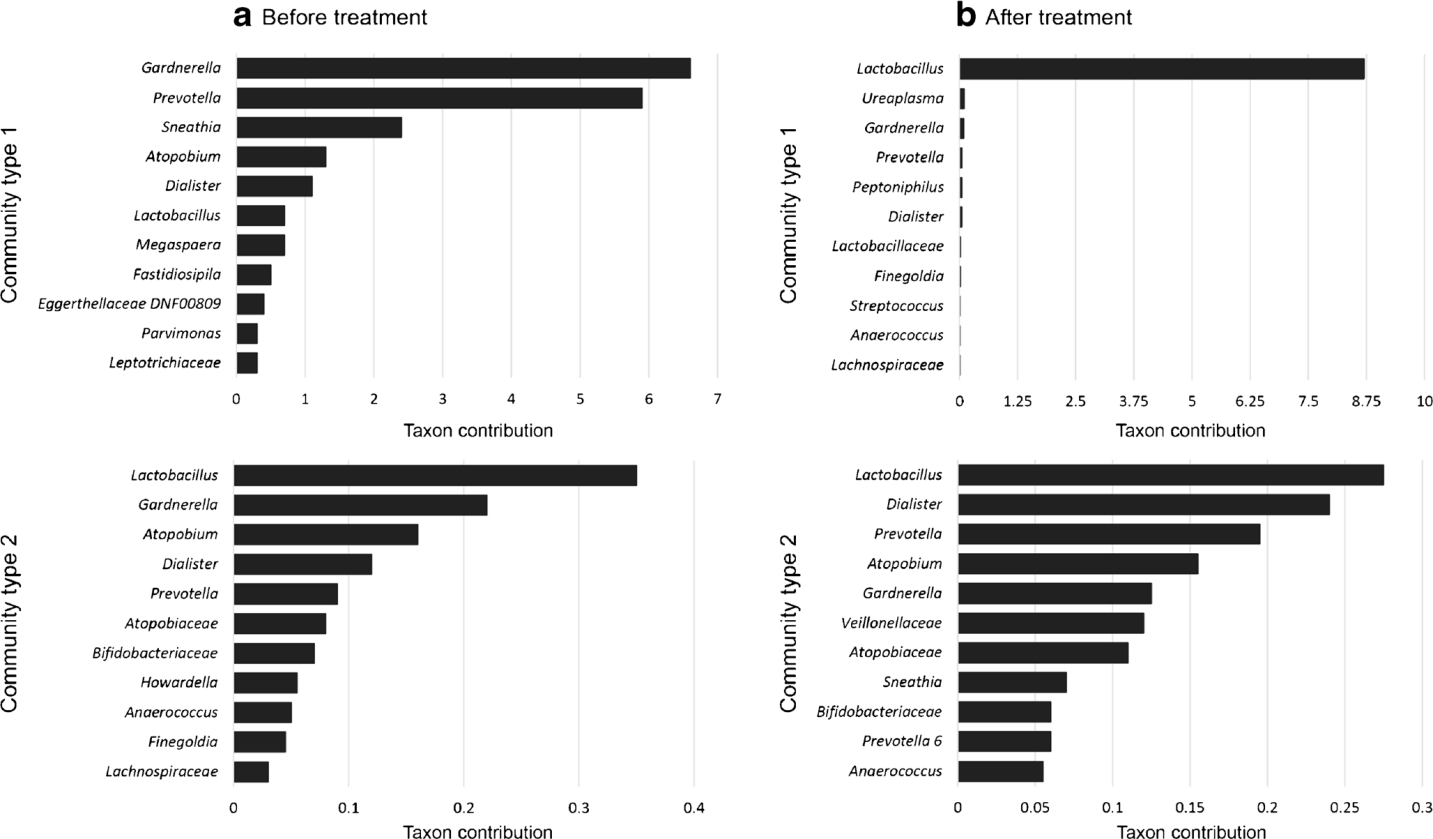
Vaginal microbiota community types before (**a**) and after (**b**) antibiotic treatment. For each community type, the 11 main driving bacterial taxa are shown

**Fig. 3:**
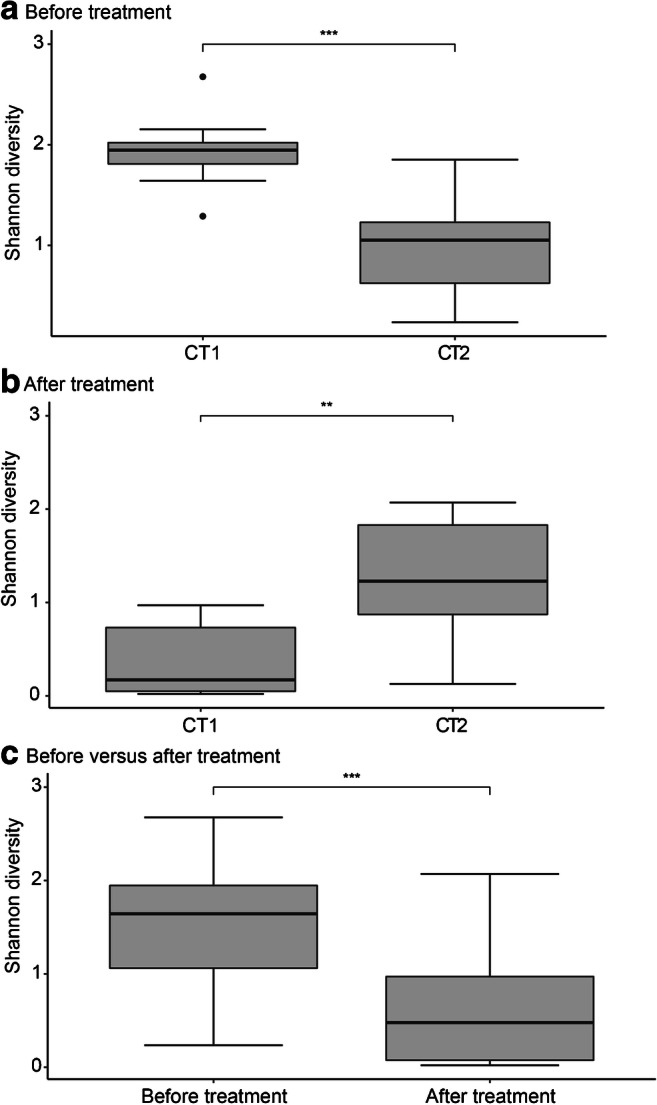
Bacterial diversity of each community type before treatment (**a**) and after treatment (**b**), and of all samples before and after treatment (**c**). Boxplots indicate the median, 25th and 75th percentile and whiskers indicate 1.5* interquartile range. *p < 0.05, **p < 0.01, ***p < 0.001. CT, community type

Before antibiotic treatment, genera *Gardnerella*, *Atopobium*, *Prevotella*, *Lactobacillus*, and *Dialister* constituted the core microbiota, and combined accounted for an average relative abundance of 71.9% (Table 2), but their abundance could vary greatly between subjects (Fig. 1a). Two community types could be identified, one driven by *Gardnerella*, *Prevotella*, *Sneathia*, and *Atopobium* (community type 1), and one driven by *Lactobacillus*, *Gardnerella*, and *Atopobium* (community type 2, Fig. 2a), suggesting *Lactobacillus*, *Prevotella*, and *Sneathia* abundances as discriminative feature of microbiota composition between patients. Bacterial diversity significantly differed between the two community types (Fig. 3a), with lower diversity in the *Lactobacillus-*driven community type. Microbiota composition before treatment was significantly associated with various parameters (Table 3), including the Nugent score, hormone-related variables (lactation, anticonception use), and BV symptomology (vaginal pH and amine odour).

**Table 2:** Core microbiota before and after antibiotic treatment. Bacterial taxa were considered part of the core microbiota when present in 75% of the samples from the specified group

Bacterial genus	Average relative abundance (fraction)
Before treatment	
*Gardnerella*	0.294
*Atopobium*	0.104
*Prevotella*	0.132
*Lactobacillus*	0.151
*Dialister*	0.038
After treatment	
*Lactobacillus*	0.608

**Table 3:** Clinical variables significantly associated with microbiota composition before and after antibiotic treatment

Variable	*R*^2^	*p* value
Before treatment		
Nugent score	0.238	0.001
Anticonception	0.146	0.008
Lactating	0.091	0.008
pH > 4.5	0.086	0.012
Amine odour	0.073	0.028
After treatment		
Nugent score	0.499	0.001
pH > 4.5	0.143	0.006

After treatment, bacterial diversity decreased (Fig. 3c) and the core microbiota solely consisted of *Lactobacillus*, constituting an average of 60.8% relative abundance (Table 2). Independent of antibiotic type (metronidazole or clindamycin), antibiotic treatment significantly decreased the relative abundance of *Atopobium* (Log2FoldChange = − 3.36, padj = 0.0388), while increasing *Lactobacillus* (Log2FoldChange = 4.04, padj = 0.0002)*.* However, *Lactobacillus* remained of low abundance in one-third of the women, whose microbiota was of individual-specific composition with high abundance of either *Gardnerella*, *Prevotella*, *Dialister*, *Escherichia-Shigella*, *Atopobium*, or *Sneathia* (Fig. 1b). These microbiota compositions were also reflected by the identification of two community types: one driven by *Lactobacillus*, and the other driven by multiple bacterial taxa (Fig. 2b), with lower diversity in the *Lactobacillus*-driven community type (community type 1, Fig. 3b). Vaginal microbiota composition after antibiotic treatment was significantly associated with the Nugent score and vaginal pH (Table 3).

These findings support the current debate on the definition of a healthy vaginal microbiota [14], since *Lactobacillus* dominance was observed in a large proportion of women with symptoms and the opposite, dominance of anaerobes, was observed in asymptomatic women. So even in a study of small subject size, as herein, heterogeneity of vaginal bacterial communities was apparent. Vaginal health status may be associated with specific *Lactobacillus* species [10], which could not be defined by the method used herein. However, several kinds of microbiota composition existed in asymptomatic women, which has been previously reported [4, 11, 15]. Vaginal microbiota composition was consistently associated with the Nugent score and vaginal pH. While the Nugent score is considered the golden standard for BV diagnosis, it is rarely used in clinical setting due to resource intensiveness [6]. Determining vaginal pH is more readily applicable; however, it most certainly simply reflects the abundance of lactic acid–producing bacteria, like *Lactobacillus*. Nowadays, PCR-based laboratory tests would be preferred for confirmation of the diagnosis [16]. Except lactation and anticonception use, vaginal microbiota composition was not associated with patient demographics and lifestyle factors, which may be due to the relatively small subject size in combination with uniformity. It has previously been reported that host genetics, ethnicity, hormonal stage (e.g. menstruation cycle, menopause, pregnancy), sexual behaviour, and hygiene practices, amongst others factors, influence vaginal microbiota composition [17–21].

After antibiotic treatment, nine women (43%) reported remaining complaints of abnormal vaginal discharge. Persisting complaints were more prevalent in women receiving metronidazole (70%) than in those receiving clindamycin (18%), which may be a result of differences in antibiotic spectrum and underlying conditions (e.g. pregnancy). To determine the potential influence of the microbiota on clinical outcome, vaginal microbiota composition before and/or after treatment were compared between patients with and without persistent complaints. The vaginal microbiota of women with persisting complaints contained a significantly higher relative abundance of *Ureaplasma* (Log2FoldChange = 8.73, pajd = 0.0008), but persisting complaints could not be associated with microbiota composition before treatment. *Ureaplasma* is a parasitic and saprophytic bacterium belonging to the Mollicutes class and is without cell wall, which results in intrinsic resistance to cell wall–targeting antibiotics like beta-lactam and glycopeptide antibiotics [22]. *Ureaplasma* is intrinsically resistant to metronidazole, but usually susceptible to clindamycin [23]. While carriage of *Ureaplasma* in the urethra, cervix, and vagina is common and generally asymptomatic, it has previously been associated with BV recurrence [24]. Treatment outcome was not associated with the identified community types after treatment as persistent complaints were reported in 50% (7/14) and 29% (2/7) of women with vaginal microbiota composition belonging to the *Lactobacillus*-driven community type one or multiple species-driven community type two, respectively.

## Conclusion

In conclusion, defining a (un)healthy vaginal microbiota state remains elusive, which challenges diagnosis and treatment of BV. Abnormal vaginal discharge and itching/irritation is most certainly not attributable to one or more specific bacteria, rather a disruption of the individual-specific mutualistic relationship of bacterial communities. Nevertheless, establishing universal markers for diagnosis and treatment of BV remains relevant. Herein, remaining complaints after treatment were more common in women who received metronidazole and were associated with increased relative abundance of the *Ureaplasma* genus, which may be considered when treatment fails.

## Data Availability

Raw sequencing data are available in the NCBI Sequence Read Archive (https://www.ncbi.nlm.nih.gov/sra) under study accession PRJNA524112.
